# A DFT study to probe homo-conjugated norbornylogous bridged spacers in dye-sensitized solar cells: an approach to suppressing agglomeration of dye molecules†

**DOI:** 10.1039/c9ra10898j

**Published:** 2020-04-17

**Authors:** Anusuya Saha, Bishwajit Ganguly

**Affiliations:** Computation and Simulation Unit (Analytical Discipline and Centralized Instrument Facility) Industrial Research Central Salt & Marine Chemicals Research Institute (CSIR-CSMCRI) Bhavnagar Gujarat-364002 India ganguly@csmcri.res.in gang_12@rediffmail.com +91-278-2567562; Academy of Scientific and Innovative Research, Council of Scientific Research, CSIR-CSMCRI Bhavnagar Gujarat-364002 India

## Abstract

This work reports a sigma-bridged framework as spacers to design new dye-sensitized solar cells. The norbornylogous bridged spacer can avoid π–π aggregation of dye molecules on the semiconductor surface in DSSCs. These sesquinorbornatrienes are known to exhibit electron propagation through the interaction of sigma and π orbitals *via* through bond (OITB) and through space (OITS) mechanisms. Density functional theory (DFT) calculations performed with these spacers and a modelled simple donor unit like *N*,*N*-dimethylamine and cyanoacrylic acid as the anchoring group showed significant results with the requisite optical parameters for DSSCs. The newly designed dyes have shown comparable or better optical properties compared to the reference dye molecule with π-conjugated thiophene spacer units. The Δ*G*_injection_, *V*_OC_ and *μ*_normal_ values calculated for the designed dyes were found to be higher than those of the reference system. The *trans*-sesquinorbornatriene system spacer (6-D) showed a *V*_OC_ of 3.3 eV, Δ*G*_injection_ of 2.4 eV and oscillatory strength (*f*) of 0.96. The total and partial density of states indicates a good communication between the valence and conduction band for the designed dyes. Transition density matrix results suggest that the exciton dissociation in the excited state is sufficiently high to overcome the coulombic attraction of the hole. These results are promising for the design of dye molecules with such scaffolds, to achieve better efficiency and to eliminate one of the major issues with π-spacer units in DSSCs.

## Abstract 2

This work reports homoconjugated norbornylogous spacers to supress agglomeration of dye molecules with improved efficiency of dye sensitized solar cells.

## Graphical abstract

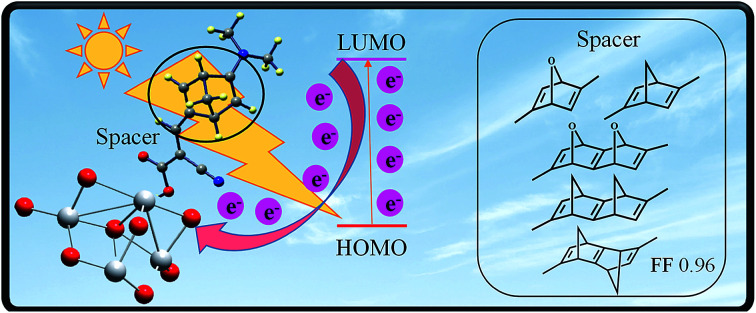

## Introduction

Dye-sensitized solar cells (DSSCs) have received attention in the last two decades. Solar cells are now being considered as an alternative renewable energy source to meet the requirement of increasing energy demand. The first Dye-sensitized solar cell (DSSC) was reported by O' Regan and Gratzel in 1991.^1^ The body of work reported with the metal complex dye molecules to achieve photovoltaic efficiency has limited application in technologies due to higher cost, limited resources and hazardous materials involved in the process.^2^ The abundance of raw materials, low-cost, flexibility of molecular design and higher molar extinction coefficient influence researchers to design and synthesize a large number of efficient organic dyes.^2^ Efforts are under way to design new organic dye molecules to augment the efficiency of DSSCs and the literature reports reveal that the power conversion efficiency has reached up to 14.6%.^3–6^

**Scheme 1:**
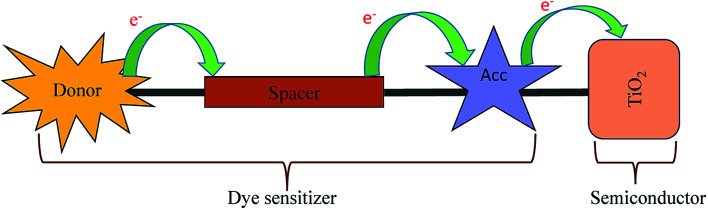
Configuration of organic donor–spacer–anchor attached to the semiconductor surface.

The organic DSSCs are comprised of donor–spacer–acceptor units anchored with the semiconductor (Scheme 1).^7^ In the 1^st^ step of DSSC, the dye sensitizer anchored on the semiconductor surface of TiO_2_ absorb the incoming light. The dye gets excited and a charge separation takes place at the interface of dye and semiconductor through the photoinduced electron injection process.^7^

The injection of an electron into the conduction band of TiO_2_ is the result of photoexcitation. The rate of electron injection can be modulated with the excited state energy of the LUMO of the dye molecule.^7^ These electrons may be recombined with the oxide of the dye sensitizer or redox electrolyte.^7^ I^−^/I_3_ is generally considered as the electrolyte in DSSCs.^8^ There are reports that the minor changes in the donor–spacer–anchoring group can change the photophysical and electrochemical properties of the dye molecules.^9^ Hence, the design of molecular sensitizers depends on the appropriate selection of donor and anchoring groups for achieving more efficient solar cells. A smaller gap in the position of HOMO and LUMO energy levels facilitates the electron injection and dye regeneration resulting in high power conversion efficiency (PCE).^10^ A strong absorption band that could cover practically all visible and near-infrared light region to maximize the light-harvesting efficiency (LHE) and a good intramolecular charge transfer (ICT) is required to enhance the efficiency of the dye-sensitized solar cell.^11^ The donor group attached to the dyes play a major role in the performance of DSSC.^11^ A variety of donor groups such as dialkylamine group, coumarins, carbazole, quinoxaline, and many others are reported as organic dye molecules and such groups have also been prepared to increase the absorption maxima near NIR region.^12^ Porphyrin and their derivatives have been exploited in DSSCs for their strong absorption ability in the visible region.^13^ Benzothiadiazole (BTD)-phenyl using as a spacer between porphyrin and anchoring group shifts the absorption ability in the NIR region.^14^ Further, the electron-donating property of bulky fluorenyl donor has been used to raise the HOMO energy levels to narrow down the HOMO–LUMO energy gap, which can enhance the *V*_OC_ values.^15^ The *N*-annulated polyaromatic systems possess efficient light harvesting properties in the solar spectrum, *i.e.*, ranges from UV-vis to NIR region. Such molecular systems can be tuned to modulate the molecular energy level to achieve better charge separation.^16^ Cyanoacrylic acid is generally used as an anchoring group due to the presence of a strong electron-withdrawing cyanide group.^11^ The strong binding ability of cyanoacrylic acid on the TiO_2_ surface facilitates the electron injection process.^17^

To enhance the performance of DSSCs – the improvements in semiconductor systems as well as in the counter electrodes have also been reported. Different concentration of polyvinylpyrrolidone (PVP) has been studied to optimize the condition to prepare TiO_2_ nanoparticles to increase the efficiency of DSSC.^18^ TiO_2_ nanofibers embedded with Al_2_O_3_ nanoparticles used as photoanode has given significant enhancement in the efficiency of DSSC.^19^ The interconnected pore structure of electrospun (PAN) with CoS nanocomposite membrane has been prepared to increase the charge transfer through redox mechanism and preventing the leaking of electrolyte in DSSCs.^20^ The replacement of costly conventional Pt electrode in DSSCs with cheaper electrode has also been reported.^21^ Nanohybrid material comprised of cobalt–nickel selenide (Co_0.50_Ni_0.50_Se) nanoparticles dispersed on graphene nanosheets (GN) has been synthesized to use as a cheaper and better counter electrode in DSSC to replace the conventional and expensive Pt electrode. Quantum dot sensitized solar cell has opened a new area for the preparation of solar cells.^21^

The spacer units help in the charge transfer from the ground state to the excited state to enhance the light absorption of DSSCs. In general, the π conjugated spacer systems have been used because of the absorption of light at a longer wavelength. However, the use of π spacers in DSSCs causes the π-stacking aggregation of the dye-sensitizer which in turn hinders the electronic propagation.^22^ There are a few reports to prevent the agglomeration of dyes in DSSCs. Spirobifluorene has been used to design three-dimensional solar cells to prevent agglomeration of the dye molecules by modulating the acceptor groups to optimize PCE.^23–27^

**Fig. 1:**
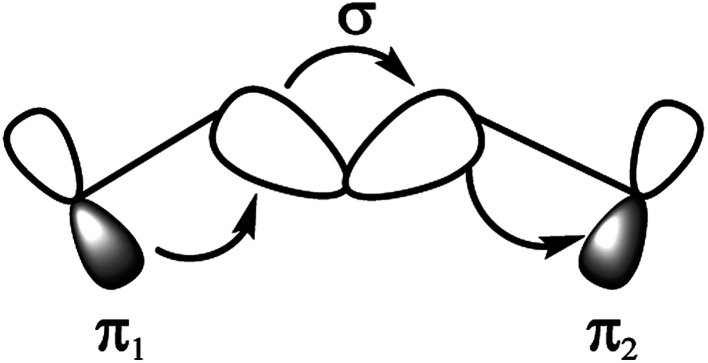
Interaction diagram representing through-bond coupling involving two degenerate π_1_ and π_2_ orbitals, interacting with the σ-orbitals of a hydrocarbon bridge.^28^

In this work, we report a new spacer to avoid the π–π aggregation of dye sensitizer molecules in DSSCs. The π spacer units have been replaced with the rigid σ-homo-conjugated framework. The three-dimensional structure of the homo-conjugated spacers eliminates the possibility of π–π aggregation. Literature suggests there are many examples revealing the long-range electron transfer (ET) process with σ-homo-conjugated systems.^17,28–30^ The electron transport in that rigid σ-homo-conjugated frameworks are suggested to be propagated through two mechanisms: (1) through orbital interaction of bonds (OITB) and (2) through space interaction of orbitals (OITS) (Fig. 1).^28,30^ In such studies, norbornylogous bridge systems are widely used for the electronic propagation processes. The sesquinorbornatrienes arranged in trans-orientation (W like bond arrangement) of σ bonds are more efficient to transfer the electron from donors to chromophores.^28^ It is suggested that the favourable orbital overlap in trans-orientation leads to through bond orbital interaction (OITB) more effectively^30–32^

The π spacers such as thiophene have been extensively used in DSSCs between the donor and anchoring moieties.^33^ Such π spacers tend to aggregate on the semiconductor surface and hence hindering the efficiency of DSSCs. We have exploited the norbornadiene and sesquinorbornatriene systems as spacers to propagate the electron transfer in DSSCs. There are reported synthetic routes for the studied norbornylogous scaffolds.^34–36^ The three-dimensional structure of sesquinorbornatriene would strictly avoid the aggregation of such dye molecules on the semiconductor surfaces. Importantly, the electron propagation should also be efficient with these spacers to achieve the performance of DSSCs. We report here for the first time the use of homo-conjugated spacers in DSSCs computationally. Further, the strategies undertaken to restrain intermolecular aggregation and charge recombination of the sensitizers are introducing alkyl chain, and bulky group or co-sensitization, and dye featuring with an encapsulated insulated molecular wire as an auxiliary donor with the π-spacers.^37,38^ These efforts are synthetically challenging and systems become heavier to manipulate to the efficiency of DSSCs.

## Computational section

The energy conversion efficiency (*η*) of a solar cell device directly relates on the open-circuit photovoltage (*V*_OC_), short circuit current density (*J*_SC_), and fill factor (FF), while inversely related with incident solar power (*P*_INC_), and is generally expressed through the following equation:^1^1
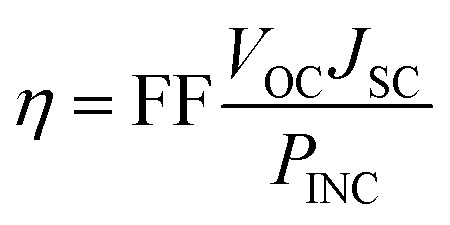


The *J*_SC_ value is determined following the equation:^1^2
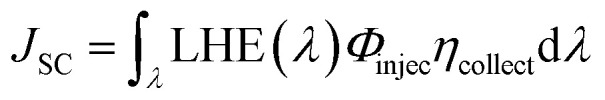
where LHE(*λ*) is the light-harvesting efficiency at a given wavelength, *Φ*_injec_ is the electron injection efficiency, and *η*_collect_ denotes the charge collection efficiency. LHE(*λ*) can be calculated with

3 LHE = 1 − 10^−*f*^

where *f* is the oscillator strength of the adsorbed dye molecule related to *λ*_max_.^1^

The rate of electron injection from the dye to the TiO_2_ conduction band is very useful for studying photovoltaic performance. The following equations are used to calculate the free energy change (in eV) for the electron injection and rejection.^39^

4 Δ*G*_injec_ = *E*^dye^* − *E*_CB_

5 Δ*G*_reg_ = *E*_I^−^/I_3_^−^_*E*_HOMO_

*E*_CB_ is the reduction potential of the conduction band of the semiconductor. *E*^dye^* is the oxidation potential of the dye after its excitation. Here we use a widely used experimental value for the conduction band *i.e.* −4.00 eV in a vacuum.^40^

The *E*^dye^* can be estimated by this equation

6 *E*^dye^* = *E*^dye^ − *λ*_max_

*λ*_max_ is the vertical transition energy of the dye and *E*^dye^ is the redox potential of the ground state.^40^

The *V*_OC_ of DSSCs can be estimated by the following equation:

7 *V*_OC_ = *E*_LUMO_ − *E*_CB_

Here, *E*_LUMO_ is the energy of the LUMO of dye, *E*_CB_ is the conduction band edge of the semiconductor substrate (here TiO_2_, −4.00 eV).^11^

The position of the band edge level can be shifted by adsorption of dipolar molecules onto the TiO_2_ surfaces. This surface modification strategy has shown improvement for the *V*_OC_ of liquid dye-sensitized solar cells. *Δ*_CB_ is the shift of the conduction band edge of the semiconductor when the dye is adsorbed on it and can be expressed as8
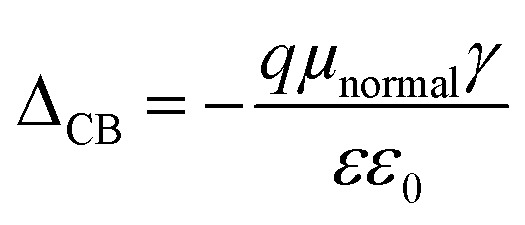
Here *ε*_0_ is vacuum permittivity and *ε* is the dielectric permittivity. The *μ*_normal_ is the dipole moment of the dye molecules which is perpendicular to the surface of the semiconductor and *γ* is the concentration of the dye on the semiconductor surface.^10^

The DFT functional Coulomb-attenuating Becke's three-parameter hybrid functional with the correlation formula of Lee, Yang, and Parr (CAM-B3LYP) provides useful accuracy for long-range orbital–orbital exchange interaction. This function is widely applicable for the polarizability of long-chain, excitation to Rydberg states and charge-transfer excitations.^41^ For this advancement in the functional CAM-B3LYP is popularly used for DSSC.^10,40^ The geometry optimizations were performed for dyes 1–6 with TiO_2_ using CAM-B3LYP DFT functional in this study. All systems were fully optimized with the 6-31G(d) basis set for the C, H, O, N atoms and effective core potential (ECP) LANL2DZ and its associated basis set for the Ti atom. All dye molecules were anchored on (TiO_2_)_4_ clusters in calculations.^2^ There a number of TiO_2_clusters in general represented as (TiO_2_)_*n*_ while *n* could be any positive integer from 1 to 38 or even more. Literature suggests cluster having even *n* are more stable than odd *n* clusters as former usually have covalent nature while the latter have ionic nature.^42^ For computational simplicity, the semi-conductor (TiO_2_)_4_ was considered here.^32^ Harmonic vibrational frequency calculations were performed and the positive frequency confirms the optimized structural minima. All the optimizations were done in the polarizable continuum model (CPCM)^43^ using tetrahydrofuran (THF) as the solvent.^44^ TD-DFT calculations were performed with the CPCM model at the CAM-B3LYP/6-31G(d) level for 50 lowest single–single excitations for simulating the absorption spectra of the isolated dyes in tetrahydrofuran (THF) solution. The *μ*_normal_ was calculated by running the optimized geometry in the gas phase attached to the (TiO_2_)_4_ cluster surface. All the calculations were done using Gaussian 09 software.^45^

The CAM-B3LYP/6-31G(d) level of theory undertaken in this study was examined with the reported.^46^ DSSC dye molecules (*Z*)-2-cyano-3-(4-(*E*)-4-(diphenylamino)styryl)phenylacrylic acid (TA-ST-CA)) (Fig. S1 in ESI†) has been computed with CAM-B3LYP/6-31G(d) using CPCM solvent model and THF as solvent to calculate the absorption maxima (*λ*_max_) and to compare with the observed results (Table S1 in ESI†). The other DFT functionals *i.e.*, B3LYP/6-31G(d), PBEPBE/6-31G(d), MPW1PW91/6-31 G(d) were also employed to compare the results with the observed UV-vis spectra of (TA-ST-CA). The calculated (*λ*_max_) values for the reported system were largely different with these DFT functionals (Table S1 in ESI†). The total density of states and the partial density of state calculations were performed using the CAM-B3LYP/6-31G(d) level of theory in the CPCM solvent model using THF as a solvent.^47^ Transition Density Matrix (TDM) was calculated using the same method to calculate the exciton dissociation in the excited state.^47^

## Results and discussion

**Scheme 2:**
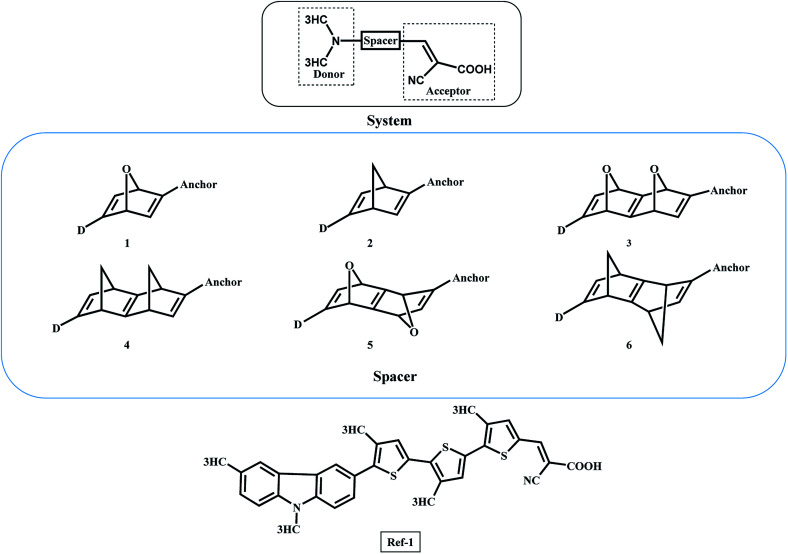
The donor (*N*,*N* dimethylamine)–spacer–anchor (cyanoacrylic acid) framework. The spacer groups 1 to 6 are studied with the same donor and anchoring group. Ref-1 is the reference DSSC system with π-spacers for comparison with the designed systems.

The general DSSC framework is constructed with a donor–spacer–anchoring group (D–S–A) and anchored on the semiconductor surface (Scheme 1). There are a number of reports available with different donor groups.^11^ Cyanoacrylic acid is commonly used as an anchoring group whereas, thiophene, substituted thiophene, and its analogous have been reported as spacer groups.^11^ This work reports the donor–spacer–anchoring group (D–S–A) as follows: *N*,*N* dimethylamine as donor molecule, homo-conjugated norbornadiene and sesquinorbornatriene systems as spacer unit and cyanoacrylic acid as anchoring group (Scheme 2). To compare the optical properties of these designed DSSCs, a reference system has also been considered containing π spacer thiophene groups (Scheme 2).

The following parameters have been examined towards the efficiency of these designed DSSCs oscillatory strength (*f*), light harvesting efficiency (LHE), free energy for electronic injection in the conduction band (Δ*G*_injec_), free energy for recombination of electron into the ground state (Δ*G*_rejction_), open-circuit voltage (*V*_OC_), and *μ*_normal_ have been calculated. The LHE is the light absorbed by the dye-sensitized semiconductor film (usually TiO_2_ film). Lambert–Beer law suggests the LHE of a cell depends on dye extinction coefficient, the attached dye concentration and the optical path length within the semi-conduction film.^32^

The photocurrent efficiency (PCE) also designated as *η* depends on short circuit voltage (*J*_SC_) and also *V*_OC_. Eqn (2) suggests *J*_SC_ is also one of the major factors for enhancing the efficiency of the cell, which depends on LHE and *Φ*_injec_ of the dye molecule. Δ*G*_injec_ is defined as the free energy change for the injection of an electron from dye excited state to the conduction band. The driving force for the electron injection from a dye to a semiconducting oxide is the energy level difference between the dyes' excited state LUMO level and the *E*_CB_ of the semiconducting oxide. The negative sign of Δ*G*_injec_ suggests the spontaneity of the process.

Dye regeneration is the essential process in which absorbed light energy converted to electrical power in DSSCs. The potential energy difference between the oxidized dyes' HOMO and the redox potential of the applied electrolyte is the driving force for the dye regeneration in DSSCs. It has been reported that an insufficient driving force for dye regeneration causes a low open-circuit voltage (*V*_OC_) and poor photocurrent generation, because of fast recombination between the injected electrons and the photooxidized dye molecules.^32^ Another factor for the higher efficiency of DSSC is *μ*_normal_. The larger the vertical *μ*_normal_ of the adsorbed dyes pointing outward from the TiO_2_ surface more enhancement in the *V*_OC_ to influence the efficiency of the dye-sensitized solar cells.^48^

The Ru containing metal-based dyes, N3, N719 are usually known to show good efficiency due to their wide absorption range from UV to near-infrared region.^7^ However, metal base dyes have demerits like leaching from the semiconductor surface and heavy metal environmental hazards as well as higher cost and limited resources.^39^ Organic dyes are alternatives to overcome such problems.

**Fig. 2:**
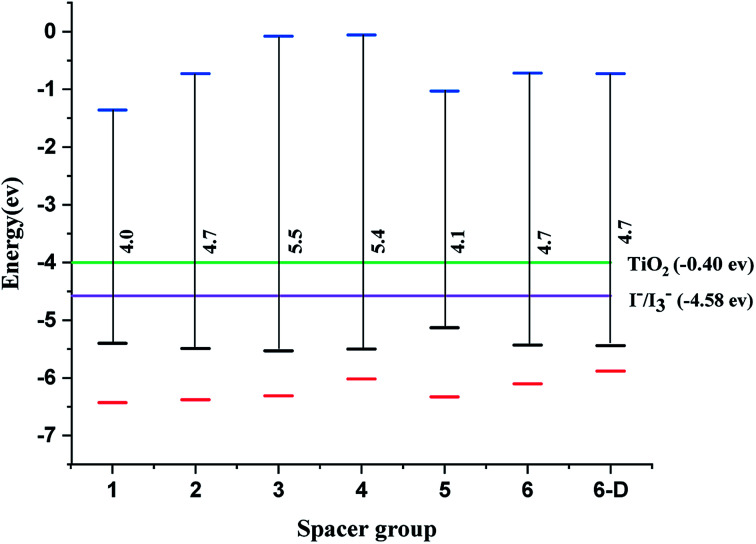
Energy of HOMO–LUMO for spacer 1–6 and 6-D calculated at CAM-B3LYP/6-31G(d) level of theory, TiO_2_ – green and the electrolyte (I_3_^−^/I^−^) – violet, black – HOMO, red – HOMO-1, blue – LUMO.

The qualifying quality for a dye in DSSC is that the dye must have the highest occupied molecular orbitals (HOMO) lower than the valence band of the semiconductor and lowest unoccupied molecular orbitals (LUMO) should be higher than the electrolyte I^−^/I_3_^−^. The system leads to redshift or bathochromic shift lowering the HOMO–LUMO gap (HLG).^11^ The studied systems show that LUMOs lie above the energy of the conduction band and the HOMOs lie above the electrolyte *i.e.* I^−^/I_3_^−^ (Fig. 2). In literature, the dyes anchored on TiO_2_ surfaces are highly conjugated and hence the HOMO–LUMO gap can be adequately modulated to improve the transition of electrons. Importantly, the homo-conjugated systems such as sesquinorbornatriene (Scheme 2) can have larger HLG and possibly leads to the *λ*_max_ values in the UV or lower visible regions. However, there are reports that strained systems can induce lower HLG with the raising HOMO level.^49^

The decrease in the HLG in strain systems can be rationalized by orbital rehybridization developing from the alteration in σ & π orbital interactions.^43^ In general, oxa-norbornadiene and oxa-sesquinorbornatriene spacers (1, 3 & 5) the –C–O bond lengths are relatively shorter than the norbornadiene and sesquinorbornatriene (2, 4 & 6) –C–C– bond lengths and hence can cause more strain than the former systems (1, 3 & 5).

For computational simplicity, we have modelled the donor molecule as *N*,*N* dimethylamine in the designed DSSCs. The studied dye molecules were compared with a calculated ref-1 system, which possesses π-conjugated thiophene spacers at the same level of theory.^10^ The donor molecule (3,9-dimethyl-9*H*-carbazole) used in ref-1 is a better donor in comparison to the *N*,*N* dimethylamine used in this study. The donor molecule (3,9-dimethyl-9*H*-carbazole) was synthetically prepared and used as a donor in several DSSCs.^44^ The Δ*G*_injec_ calculated for the designed dyes anchored on the TiO_2_ surface is given in Table 1. The calculated Δ*G*_injec_ has shown the negative values indicating the electronic injection from the excited state LUMO to the semi-conduction band is spontaneous in nature. Interestingly, all the studied dyes 1–6 yields higher Δ*G*_injec_ values than the ref-1 system. The Δ*G*_injec_ values compared for 1 and 2 suggests that the former has 0.2 eV higher than the latter case, however, 3 and 4 with extended sesquinorbornatriene systems showed comparable Δ*G*_injec_ values of 1.4 eV. Among all the studied spacer systems, 5 has the highest Δ*G*_injec_ value of 3.5 eV. The Δ*G*_injec_ values for all the studied systems are higher than that of ref-1 suggests that homo-conjugated spacers would be more efficient than the conjugated thiophene spacers. To further examine the improvement in DSSCs with homoconjugated spacers, additional calculations have been performed with the reported dye TA-ST-CA using planar styrene spacer.^46^ The calculated Δ*G*_injec_ value for TA-ST-CA is 1.7 eV (Table S2 in ESI†). The calculated result suggests that the Δ*G*_injec_ value is lower or comparable to the designed homoconjugated spacer systems (1–6). Therefore the result suggests that the new spacers can transport the exciton as efficiently or even better as compared to the π-spacers like thiophene or styrene. The use of homoconjugated spacers can avoid the number of π-conjugated spacers in the dye molecule to achieve similar or higher efficiency of DSSCs. The reduction in the spacer units will be useful for the synthetic manipulations.

**Table 1:** Calculated values for *f*, LHE, *λ*_max_, *μ*_normal_, Δ*G*_injec_, Δ*G*_rejection_, *V*_OC_ for the system (1–6 and 6-D) using CAM-B3LYP/6-31G(d) level of theory and CPCM solvation model in THF solvent

System no.	*f*	LHE (10^−1^)	*λ*_max_ (nm)	*μ*_normal_ (debye)	Δ*G*_injec_ (eV)	Δ*G*_rejection_ (eV)	*V*_OC_ (eV)
Ref-1	1.76	9.8	484.0	10.75	1.2	0.81	—
1	0.47	6.61	279.7	11.72	3.0	0.82	2.6
2	0.81	8.45	289.4	15.13	2.8	0.91	3.3
3	0.09	1.78	430.3	13.35	1.4	0.95	3.9
4	0.08	1.73	422.7	13.86	1.4	0.93	3.9
5	0.20	3.69	269.7	18.45	3.5	0.55	3.0
6	0.38	5.78	280.9	21.13	3.0	0.84	3.3
6-D	0.96	8.9	324.1	49.28	2.4	0.86	3.3

The Δ*G*_rej_ is the free energy difference between the electrolyte and the HOMO of the dye molecules. It signifies the ability of the dye to get reduced by the electrolyte used in the DSSC. If the Δ*G*_rej_ value is lower, that suggests higher recombination efficiency and the negative sign indicates the reaction is spontaneous in nature. HOMO of all dye molecules has higher values than the electrolyte (I^−^/I_3_^−^). Spacer 5 shows much lower Δ*G*_rej_ values than the reference system showing that this dye molecule can have better or comparable efficiency as compared to π-spacer units in DSSC (Table 1). The experimentally reported dye shows the calculated value for Δ*G*_rej_ is 0.84 eV. It is to note that 5 possesses the higher HOMO energy than the other spacer groups leading to the lowest Δ*G*_rej_ value. The oxa-norbornadiene and oxa-sesquinorbornatriene systems (1, 3 & 5) have better Δ*G*_rej_ values as compare to norbornadiene and sesquinorbornatriene systems (2, 4 & 6).The former systems (1, 3 & 5) possess more strain due to the shorter –C–O bond length and as a result, the HOMO energy levels of them increase. The energy gap between the HOMO of oxa-sesquinorbornatriene systems and the oxidation state of electrolyte decreases and hence the former systems show better Δ*G*_rej_ values than the latter systems.

**Fig. 3:**
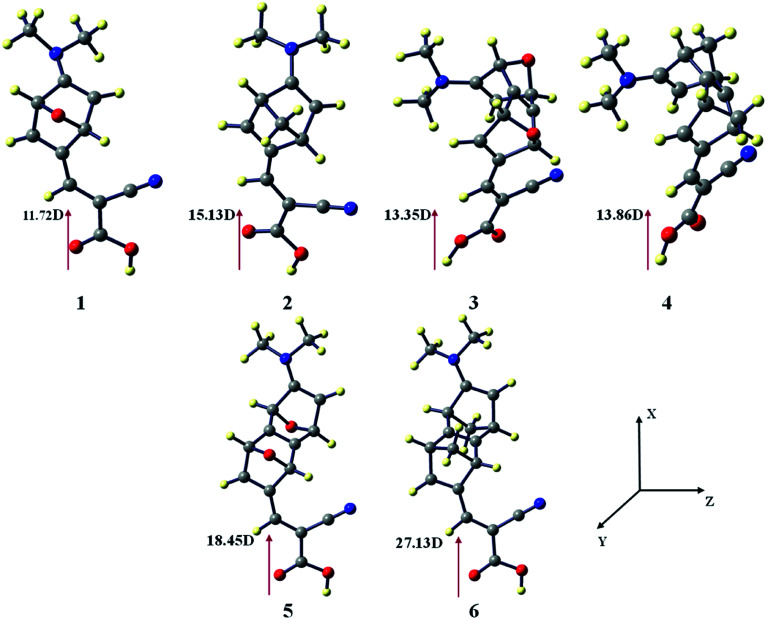
Vertical dipole moments of dye 1–6 at the CAM-B3LYP/6-31G(d) level of theory using the CPCM solvent model in THF solvent. The TiO_2_ surface is parallel to the *yz* plane.

There is a correlation between the dipole moment (*μ*_normal_) of the dye anchored with the semi-conductor and the *V*_OC_. A dye with larger *μ*_normal_ value will induce a significant change in *V*_OC_ (Fig. 3).

We have calculated the vertical dipole moment of the dye attached with the (TiO_2_)_4_ semi-conductor surface (Table 1). The calculated results show all the spacers attached with dye molecules and the TiO_2_ surface have a greater dipole moment than the reference system (Fig. 2). Dye 6 shows the highest *μ*_normal_ value in the studied systems (Table 1).

The LHE is directly related to oscillatory strength *f* and the increased LHE enhances the *J*_SC_ value resulting in the efficiency of the solar cell. The TD-DFT calculations for all the spacer groups attached with dye molecule anchored on the TiO_2_ surface suggest that the *f* are within the range 0.17 to 0.89 (Table 1) and LHE is within the range of 0.17 to 0.84. The experimentally reported system (TA-ST-CA) has oscillatory strength of 1.8 and LHE of 0.98. The LHE for the dye TA-ST-CA and designed dyes 6 with a better donor group (6-D) is comparable.

**Fig. 4:**
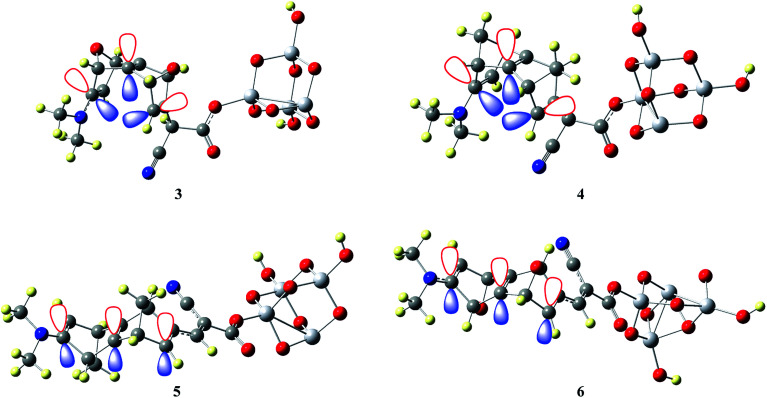
π-orbital arrangement of the sesquinorbornatriene (3–6) spacer groups.

The calculated results suggest that sesquinorbornatriene systems with trans-orientation (5 & 6) yield general better optical properties than the *syn*-orientation (3 & 4). It appears that the dyes 3 & 4 suffers from severe steric strain consequently the sequential π orbitals of the system orients in orthogonal fashion and hence lowers the propagation of electrons from donor molecule to the semi-conductor compared to 5 & 6 (Fig. 4).

**Fig. 5:**
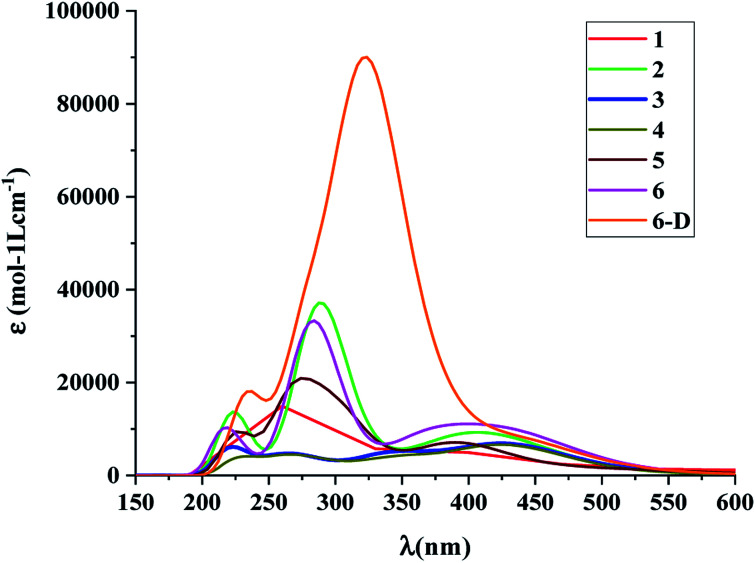
Absorption spectra of the dye molecules 1–6 and 6-D at CAM-B3LYP/6-31G(d) level of theory using the CPCM solvent model in THF solvent.

The calculated absorption spectra of the DSSCs with the spacer groups 1–6 are summarized in Table 1 and Fig. 5. The absorption spectra were calculated employing the TD-DFT method.

**Fig. 6:**
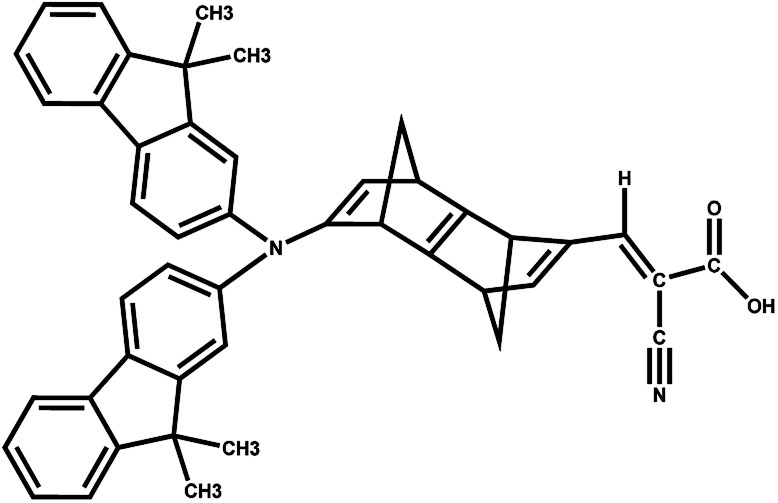
DSSC system 6 attached with better donor *N*-(9,9-dimethyl-9*H*-fluorene-2-yl)-9,9-dimethyl-*N*-phenyl-9*H*-fluorene-2-amine (6-D).

The results suggest that 2 has the highest *f* with a *λ*_max_ value of 289.4 nm (Fig. 5). However, dye 6 also possesses a better *f* value in the UV-vis region as well as in the lower visible region (Fig. 5). The *μ*_normal_ value for spacer 6 is highest among all dyes. The donor molecule used in the designed DSSCs is the simple *N*,*N*-dimethylamine. The donating ability of the donor molecule can be enhanced with larger donor groups. The Δ*G*_rej_ value for dye 5 is lowest among the studied systems, however, the calculated *V*_OC_, *μ*_normal_, *f*, LHE and *λ*_max_ values for dye 6 is relatively better than that of dye 5. Therefore, the calculations have been performed with a much better donor molecule *i.e.*, *N*-(9,9-dimethyl-9*H*-fluorene-2-yl)-9,9-dimethyl-*N*-phenyl-9*H*-fluorene-2-amine attaching with the spacer 6 (Fig. 6). The *λ*_max_ value is red-shifted about ∼43 nm for spacer 6 when it is attached to a more efficient organic donor molecule. The oscillator strength *f* also increases and the LHE value is comparable with that of the ref-1. Further, some additional calculations have been performed to compute *V*_OC_, Δ*G*_injec_, and Δ*G*_rej_ for the experimentally reported (TA-ST-CA) system (Table S2 in ESI†). The results suggest that in general, the values for the designed systems (1–6) are better or comparable with respect to the reported system (Table 1).^46^

These results reveal that the homo-conjugated spacers can also be an alternative of π-spacer units to attain higher efficiency with the proper choice of donor molecules. The intervention of smaller homo-conjugated systems as spacers can avoid the tedious synthetic routes and strategies to avoid designing new dyes to achieve better efficiency of DSSCs.^34–36^ The higher basis set 6-311+G(d) with CAM-B3LYP was also employed to examine optical properties of 6 and the calculated results suggest that the Δ*G*_injec_, Δ*G*_rej_, *V*_OC_ values are relatively superior with 6-31G(d) basis set (Table S3 in ESI†).

**Fig. 7:**
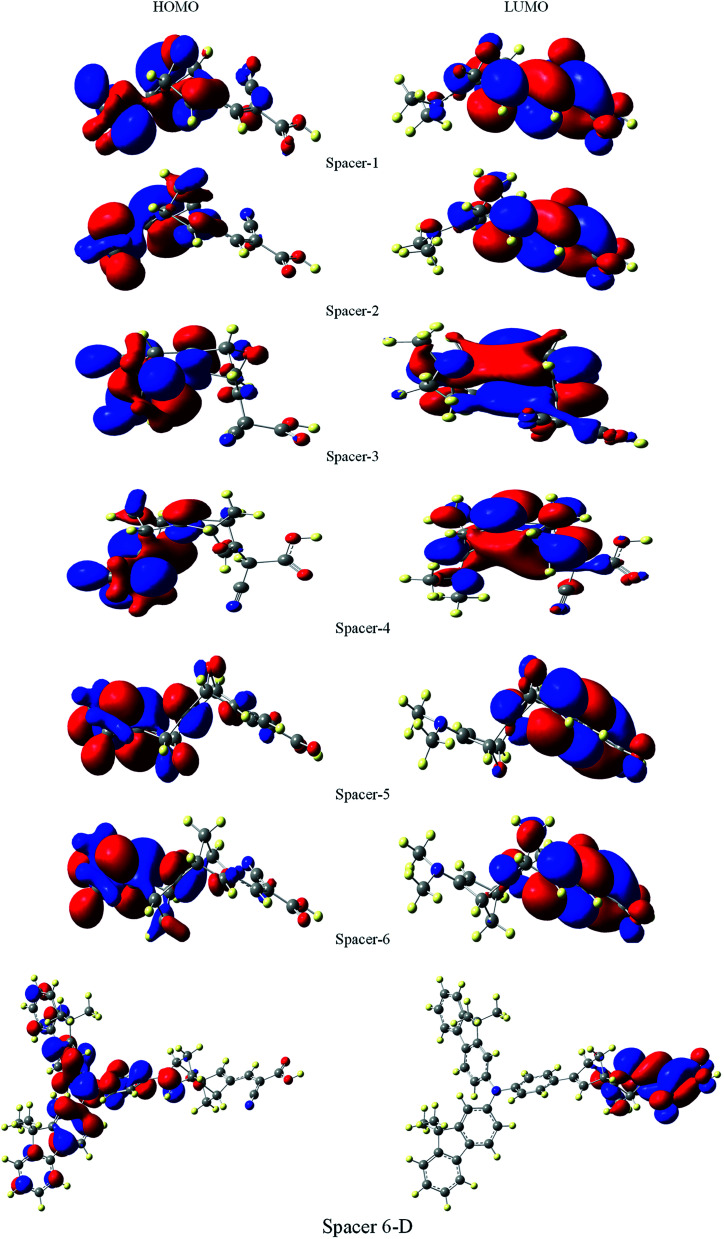
Frontier molecular orbitals of dyes 1–6 and 6-D at the B3LYP/6-31G(d) level of theory using the CPCM solvent model in THF solvent.

**Fig. 8:**
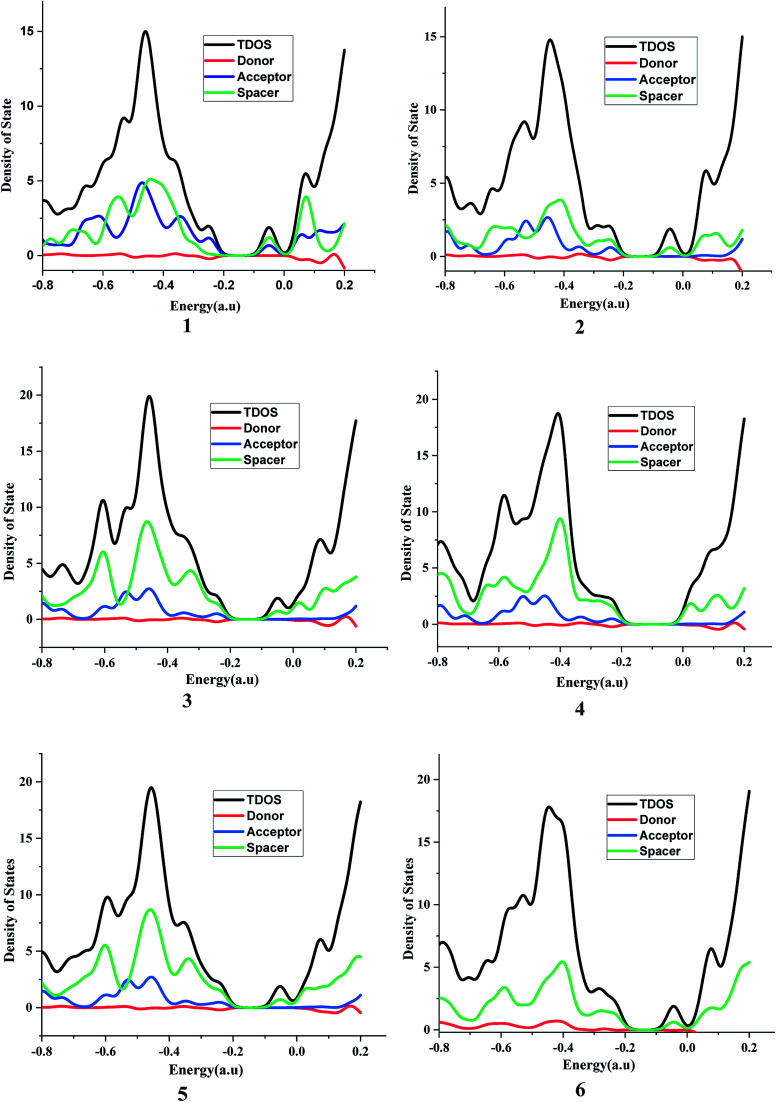
Graph for total density of states and partial density of states in CAM-B3LYP/6-31G(d) level of theory. Black – total density of states, red – PDOS for donor, green – PDOS for spacer and blue – PDOS for anchor.

It is suggested that for efficient electron injection into anode the HOMO should be localized over the D–S part and the lowest unoccupied molecular orbital (LUMO) of the dye should be localized near the anchoring group and above the conduction band edge of the semiconductor electrode (here TiO_2_).^39^ The HOMO–LUMO pictures of spacer group 1–6 and 6-D are given in Fig. 6 suggests that for all the systems HOMO orbitals are localized over D–S–A part whereas the LUMO orbitals are localized over the S–A part. These FMOs suggest that all the dyes meet the criteria to propagate electron from the donor group to the semi-conductor group (Fig. 7). To support the FMO diagram, the total density of states and partial density of states (PDOS) have been calculated for all the studied dyes in CAM-B3LYP/6-31G(d) in THF using the CPCM solvent model.^23–25^ The studied dye molecules (1–6) have been divided into three parts donor, spacer and acceptor units (Scheme 2). In PDOS spectra distribution pattern of donor, spacer and acceptor units are represented by red, green and blue lines, respectively (Fig. 8). The negative values along the *x*-axis show the valence band or the HOMO whereas the positive values indicate the conduction band or the LUMO.^50,51^ The distance between the valence band and the conduction band is indicated as band gap.^50^ The partial density of states for spacer units exhibit by green line increases by changing the spacer group from norbornadiene to sesquinorbornatriene. The DOS analyses corroborate well with the FMO results for these dye molecules.

Intermolecular charge transfer is useful in evaluating the dissociation of excitons in free charges. An effective excitation separation of donor materials can lead to increase photo generation charge carriers which improve the *J*_SC_ & FF values of DSSCs.^50^ Transition density matrix estimates the nature of transition and the TDM map can be employed to probe the possibility of excitation escaped from coulomb attraction.^24–27^ The TDM absorption and emission are computed for S1 state in the same environment. TDM method is important to estimate electronic excitation interaction between different parts of dyes in excited state and hole localization. Hydrogen atoms have a very nominal contribution in transition and neglected.^50^

**Fig. 9:**
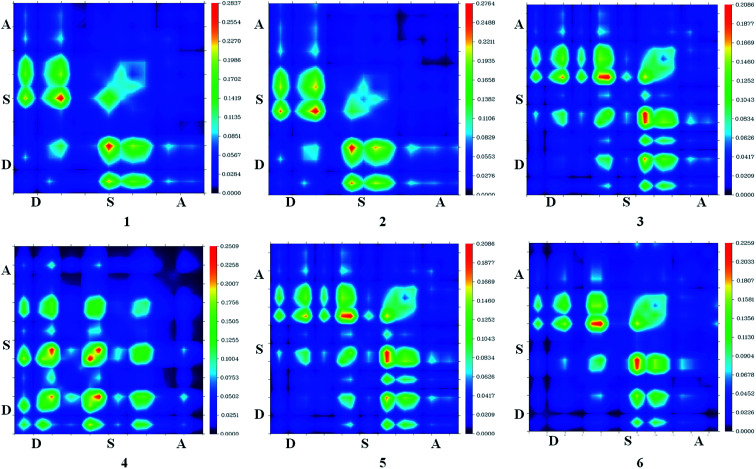
Transition density of states map for dye 1–6 for S1 excitation state using CAM-B3LYP/6-31G(d).

In this study, the studied molecules have been divided into donor (D), spacer (S), and acceptor (A) parts (Scheme 2). The plot (Fig. 9) suggests that the electron coherence for the designed molecules partially is available on the diagonal of the donor and the spacer units and significantly enhanced with sesquinorbornatriene (6) on the acceptor unit.^50^ Importantly, the coefficient of electron–hole correlation is in the order of 2 > 1 > 3 > 4 > 5 > 6. This order suggests the coupling of electron and hole in 6 is possibly very weak indicating easier excitation dissociation as compared to the other studied dyes. However, the results illustrate that excitation in all the dyes may dissociate easily owing to their weak electron–hole correlation coefficient, especially in the case of 6 indicating better charge transfer as compared to the dye molecules. Therefore, it appears that the designed spacers can effectively improve the *J*_SC_ since the exciton can effectively escape from coulomb attraction.

## Conclusion

In this work, we first report the role of the σ-homo-conjugated framework as a spacer group in dye-sensitized solar cells. The π-conjugated thiophene and its homologs have been generally used as spacer groups in DSSCs. Nonetheless, these thiophene moieties being mostly planners suffer from π-agglomeration retarding the electronic propagation in DSSCs. The σ-homo-conjugated spacer units have been introduced in DSSCs to avoid this π-aggregation on the semiconductor surface. The three-dimensional structure of this spacer group can prevent the π-agglomeration of the dye molecules on semiconductor surfaces. The norbornylogous systems allow propagating the electrons through bond interaction (OITB) as well as through space interactions (OITS). The designed new dye molecules with sesquinorbornatrienes as spacer groups have shown comparable or better optical properties compared to the reference dye molecule with π-conjugated thiophene spacer units. The *trans*-sesquinorbornatriene systems (5 & 6) give improved optical properties compared to their corresponding *syn*-orientated systems (3 & 4). It is presumed that the through space electronic propagation is better for the *trans*-sesquinorbornatriene systems due to the favourable π orbital alignment. The PDOS plots suggest the gap between valence band and conduction band decreases from norbornadiene to sesquinorbornatriene systems. The transition density matrix map suggests excited phonons will escape the coulombic attraction to enhance the charge transfer from donor to acceptor for all the dyes and sesquinorbornatriene molecule (6) shows relatively better charge transfer possibility. The dye molecule 6, when attached with a better dye (6-D), shows significant improvement in results. The Δ*G*_injection_ value of 3.0 eV is way better as compared to the ref-1 thiophene system and comparable to experimentally reported system (TA-ST-CA). The Δ*G*_rej_ value of 6-D is comparable to that of ref-1 and TA-ST-CA. The three-dimensional homo-conjugated spacers can be employed is synthesizing the dye molecules to achieve improved efficiency of solar cells. The efforts are underway to broaden the absorption spectrum using such homo-conjugated spacers in DSSCs.

## Conflicts of interest

There are no conflicts to declare.

## Supplementary Material

RA-010-C9RA10898J-s001
