# Supplementary material for: A DFT study to probe homo-conjugated norbornylogous bridged spacers in dye-sensitized solar cells: an approach to suppressing agglomeration of dye molecules
Source: RSC Adv. 2020 Apr 17;10(26):15307–19. doi: 10.1039/c9ra10898j (PMC9052607; doi:10.1039/c9ra10898j)
Supplement: RA-010-C9RA10898J-s001 [file RA-010-C9RA10898J-s001.pdf]

Electronic Supplementary Material (ESI) for RSC Advances.
This journal is © The Royal Society of Chemistry 2020

A DFT Study to Probe Homo-conjugated Norbornylogous Bridged Spacers in Dye-sensitized Solar cell: An Approach to Supress Agglomeration of Dye Molecules

Anusuya Saha[a], Bishwajit Ganguly[a,b*].

[a]Computation and Simulation Unit (Analytical Discipline and Centralized Instrument Facility) Industrial Research Central Salt & Marine Chemicals Research Institute (CSIR-CSMCRI), Bhavnagar, Gujarat-364002, India.

[b]Academy of Scientific and Innovative Research, Council of Scientific Research, CSIR-CSMCRI, Bhavnagar, Gujarat, India- 364002.

**Corresponding Author. Fax: (+91)-278-2567562, E-mail: ganguly@csmcri.res.in; gang_12@rediffmail.com*

An experimental system was computed using different DFT methods. Those computed values were compared and the method gives closest value to the experimental data was used for further study.

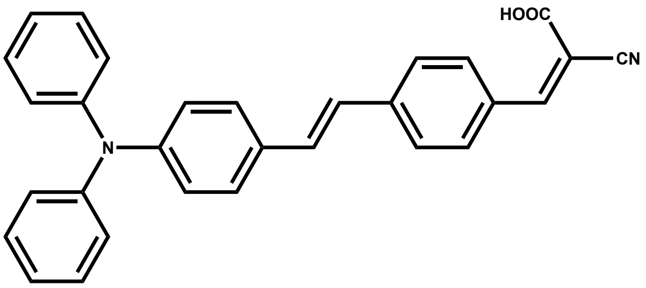

Figure S1. Structure for (Z)-2-cyano-3-(4-((E)-4-(diphenylamino)styryl)phenyl)acrylic acid (TA-ST-CA), the experimental system [*Chem. Commun.*, 2007, **46**, 4887–4889].

Table S1. UV-Visible absorption (λ_{max}) for TA-ST-CA in different DFT methods using 6-31G(d) basis set and CPCM solvent model with THF solvent.

Experimental	B3LYP/6-31G(d)	PBEPBE/6-31G(d)	MPW1PW91/6-31G(d)	CAM-B3LYP/6-31G(d)
386 nm	584 nm	479 nm	546 nm	411 nm

The structure of TA-ST-CA could not be optimized with WB97XD/6-31G(d) level due to failure of convergence criteria. Hence, this value is not incorporated in the manuscript.

Table S2. Calculate values of λ_{max}, HOMO, LUMO, ΔG_{inject}, $\Delta G_{rejection}$, V_{OC} for system TA-ST-CA with CAM-B3LYP/6-31G(d) level of theory.

System	λ_{max}	HOMO	LUMO	HOMO-LUMO gap	ΔG_{inject}	$\Delta G_{rejection}$	V_{OC}
TA-ST-CA	411	-5.4	-1.25	4.17	1.7	0.84	2.8

CAM-B3LYP/6-31G(d) level of theory was employed to calculate an experimentally reported system (TA-ST-CA) [*Chem. Commun*., 2007, **46**, 4887–4889]. The experimentally reported absorption maxima (λ_{max}) for TA-ST-CA is 398 nm and calculated value was observed to be 411 nm. The HOMO energy level of the system was experimentally determined from the oxidation potential value w.r.t ferrocene using Ag/AgCl as reference electrode in acetonitrile electrode. The calculated HOMO energy (5.4 eV) of the system is in close agreement with the experimentally observed HOMO (5.2 eV). The calculated LUMO value of the system is 1.25 eV which is lower than that of the experimentally reported LUMO (2.8 eV). It is to note that experimentally the LUMO was calculated from the absorption edge of absorption spectra and HOMO [*Chem. Commun*., 2007, **46**, 4887–4889]. Therefore, the variation in the LUMO result may be expected.

Table S3. Calculated values for f, LHE, λ_{max}, ΔG_{injec} , $\Delta G_{rejection}$, V_{OC} for the system 6 using CAM-B3LYP/6-311+G(d) in THF solvent and CPCM solvent model.

System	λ_{max}	f	LHE	HOMO	LUMO	HOMO-LUMO gap	ΔG_{inject}	$\Delta G_{rejection}$	V_{OC}
6	292.84	0.49	0.68	5.7	1.07	4.63	1.5	1.1	2.9

Coordinates for:

system 1:

C	0	-7.01065200	0.30026200	0.05140500
C	0	-6.84302600	-0.96599900	-0.83183900
C	0	-5.37123600	-1.14946000	0.68138500
C	0	-6.09063800	0.15876700	1.01922100
C	0	-5.51029400	-0.80289800	-1.57179100
H	0	-5.38977600	-0.57874700	-2.62393000
C	0	-4.56472100	-0.92787100	-0.62898600
H	0	-7.69522600	-1.30607300	-1.41540300
H	0	-4.87342700	-1.67403400	1.49146300
C	0	-3.15244500	-0.76576200	-0.83831100
H	0	-2.82606300	-0.67757300	-1.87164500
C	0	-0.73586100	-0.49363000	-0.40122700
C	0	-2.15475100	-0.68625400	0.06903700
O	0	-0.48922800	-0.42694000	-1.60050800
O	0	0.12790700	-0.41301400	0.56434200
N	0	-7.82359600	1.35811200	-0.28154500
C	0	-9.13757500	1.08356300	-0.84379300
H	0	-9.85195500	0.72170900	-0.08928200
H	0	-9.53561100	2.00279300	-1.28047100
H	0	-9.06818500	0.34007300	-1.63869200
C	0	-7.78685100	2.48226300	0.63439100
H	0	-8.34756800	3.31621300	0.20626000
H	0	-8.21829100	2.23580400	1.61682900
H	0	-6.75130100	2.79813100	0.78386100
C	0	-2.37179500	-0.75256300	1.48140500
N	0	-2.55514800	-0.80318200	2.62422800
O	0	4.28325700	2.12861200	1.11530100

Ti	-1	4.16515900	0.19157900	1.65151600
Ti	-1	2.04305900	-0.07041700	0.24912900
O	-1	2.42023600	-1.83948200	-0.74393100
O	-1	2.33768400	-0.01872600	2.21127500
O	-1	1.92471400	1.78586200	-0.64710600
O	-1	5.09429900	-1.61479100	1.21489500
Ti	-1	4.34636300	-1.47444100	-0.64177900
O	-1	5.47032000	-2.26003000	-2.06992600
Ti	-1	3.88356000	1.94048300	-0.56815500
O	-1	4.73705800	3.05657000	-1.96319300
O	-1	4.07816500	0.22025600	-0.20259800
H	-1	5.12550100	-2.00183100	-2.92761400
H	-1	4.45941400	2.75303900	-2.83073800
H	0	-5.80249000	0.83859900	1.80701800
O	0	-6.46364300	-1.92502000	0.15301800

System 2:

C	-7.25528700	-0.32036300	0.14203800
C	-7.12472700	1.03098800	-0.59926900
C	-5.51380700	-0.31094600	-1.38077400
C	-6.28919500	-1.12302600	-0.33408300
C	-5.79746700	1.64042200	-0.17159000
H	-5.68282000	2.52817400	0.44010900
C	-4.81230800	0.84500700	-0.63344900
H	-7.99337200	1.68671400	-0.55923100
H	-4.89608000	-0.88440600	-2.06725700
C	-3.42166400	1.07213500	-0.35572500
H	-3.19005800	1.99021800	0.17957100
C	-0.98085400	0.79636000	-0.21403700

C	-2.33614500	0.31652000	-0.64298200
O	-0.84150400	1.85473100	0.37974000
O	-0.00507200	-0.01266000	-0.53791400
N	-8.25001100	-0.56232600	1.07382200
C	-8.55458100	0.49409700	2.02708300
H	-9.51638000	0.28356400	2.50269500
H	-7.79185000	0.57984800	2.81684700
H	-8.63163000	1.45671600	1.51992200
C	-8.22991700	-1.88698400	1.66252200
H	-7.34735200	-2.04970300	2.30185400
H	-9.12864200	-2.02764100	2.26807700
H	-8.22137700	-2.63930500	0.86969800
C	-2.39411800	-0.93958800	-1.32358900
N	-2.42708000	-1.95992000	-1.87132100
H	-6.02421800	-2.12063000	-0.01266800
H	-7.44318500	-0.14433200	-2.45652200
O	2.69844500	-1.52457300	-0.82051400
Ti	1.87536600	-0.06049700	-0.13795500
Ti	3.71815400	-0.06871900	1.96073400
O	4.60493900	1.48065700	1.46898800
O	1.90041100	-0.09283800	1.67769100
O	4.60683900	-1.60839600	1.44487400
O	2.69957500	1.41144200	-0.81703600
Ti	4.47254200	1.47052100	-0.33205400
O	5.30312100	2.98613000	-0.92176100
Ti	4.48391300	-1.58738300	-0.35640200
O	5.29553000	-3.10057700	-0.97799100
O	5.25276200	-0.05686900	-0.99199300
H	5.67280200	3.70265200	-0.38736900
H	5.48607200	-3.90983000	-0.48328100

C	-6.68891700	0.49139400	-1.98683600
H	-6.37815500	1.28490900	-2.67358200

System 3:

C	4.65248400	1.80774200	-0.54873400
C	3.96819400	1.31233000	-1.81925700
C	2.46805300	1.22419100	-0.29251200
C	3.72036200	1.74544700	0.40276300
C	3.68567000	-0.19768000	-1.62251900
H	4.20848600	-1.00311600	-2.12043400
C	2.75729900	-0.25817700	-0.67587300
H	4.34608900	1.62230100	-2.79120500
H	1.48567300	1.43595800	0.11890600
C	5.98083500	1.72220100	0.19635300
H	6.87433600	2.14513300	-0.25675200
C	4.48356600	1.61316400	1.72049900
H	4.01392400	1.95573800	2.64091100
C	6.07064200	0.27106800	0.76779300
C	5.14363300	0.22232200	1.73976700
H	4.83362100	-0.60639700	2.35858400
C	2.12395400	-1.44005700	0.00164300
H	2.13178100	-1.26809100	1.08666600
C	0.63537800	-1.62526100	-0.38497900
C	2.87796800	-2.67132900	-0.25090100
O	0.21711300	-2.69027900	-0.79554400
O	-0.06011500	-0.54703600	-0.18768100
N	3.50532000	-3.62154400	-0.44207300
N	6.85191200	-0.71215000	0.21618000
C	8.15047300	-0.36592700	-0.34102500

H	8.90379700	-0.16496300	0.43506100
H	8.50442200	-1.19711400	-0.95532100
H	8.07200400	0.51199800	-0.98318000
C	6.79182900	-2.00458500	0.87305100
H	7.35286000	-2.73466000	0.28590700
H	7.21012800	-1.97372000	1.89059300
H	5.75237200	-2.33645400	0.93413500
O	-4.85798000	1.32968000	-1.37476800
Ti	-4.26699000	-0.40551100	-1.67526200
Ti	-1.96360800	-0.13078200	-0.20969400
O	-2.63973200	-1.18460800	1.13454200
O	-2.45153100	-0.52932500	-1.91934900
O	-2.39888800	1.56995800	0.22787500
O	-5.10428700	-1.57209500	-0.49907500
Ti	-4.45330600	-1.12162500	1.12457500
O	-5.15958600	-2.23907900	2.39044200
Ti	-4.22934200	1.81365900	0.24303300
O	-4.67255000	3.54913700	0.61380800
O	-4.87428500	0.64560600	1.47470100
H	-4.76525000	-2.39191600	3.26131200
H	-4.58578400	3.95442700	1.48898200
O	2.64825000	1.83820800	-1.57960400
O	5.62737500	2.43329900	1.38619900

System 4:

C	4.65248400	1.80774200	-0.54873400
C	3.96819400	1.31233000	-1.81925700
C	2.46805300	1.22419100	-0.29251200
C	3.72036200	1.74544700	0.40276300

C	3.68567000	-0.19768000	-1.62251900
H	4.20848600	-1.00311600	-2.12043400
C	2.75729900	-0.25817700	-0.67587300
H	4.34608900	1.62230100	-2.79120500
H	1.48567300	1.43595800	0.11890600
C	5.98083500	1.72220100	0.19635300
H	6.87433600	2.14513300	-0.25675200
C	4.48356600	1.61316400	1.72049900
H	4.01392400	1.95573800	2.64091100
C	6.07064200	0.27106800	0.76779300
C	5.14363300	0.22232200	1.73976700
H	4.83362100	-0.60639700	2.35858400
C	2.12395400	-1.44005700	0.00164300
H	2.13178100	-1.26809100	1.08666600
C	0.63537800	-1.62526100	-0.38497900
C	2.87796800	-2.67132900	-0.25090100
O	0.21711300	-2.69027900	-0.79554400
O	-0.06011500	-0.54703600	-0.18768100
N	3.50532000	-3.62154400	-0.44207300
N	6.85191200	-0.71215000	0.21618000
C	8.15047300	-0.36592700	-0.34102500
H	8.90379700	-0.16496300	0.43506100
H	8.50442200	-1.19711400	-0.95532100
H	8.07200400	0.51199800	-0.98318000
C	6.79182900	-2.00458500	0.87305100
H	7.35286000	-2.73466000	0.28590700
H	7.21012800	-1.97372000	1.89059300
H	5.75237200	-2.33645400	0.93413500
O	-4.85798000	1.32968000	-1.37476800
Ti	-4.26699000	-0.40551100	-1.67526200

Ti	-1.96360800	-0.13078200	-0.20969400
O	-2.63973200	-1.18460800	1.13454200
O	-2.45153100	-0.52932500	-1.91934900
O	-2.39888800	1.56995800	0.22787500
O	-5.10428700	-1.57209500	-0.49907500
Ti	-4.45330600	-1.12162500	1.12457500
O	-5.15958600	-2.23907900	2.39044200
Ti	-4.22934200	1.81365900	0.24303300
O	-4.67255000	3.54913700	0.61380800
O	-4.87428500	0.64560600	1.47470100
H	-4.76525000	-2.39191600	3.26131200
H	-4.58578400	3.95442700	1.48898200
O	2.64825000	1.83820800	-1.57960400
O	5.62737500	2.43329900	1.38619900

System 4:

C	4.71372400	1.89552000	-0.62144000
C	4.02695600	1.41638300	-1.89407400
C	2.50785700	1.33804500	-0.22140900
C	3.81413800	1.85324200	0.36640300
C	3.67428800	-0.07057400	-1.66465300
H	4.13841900	-0.90265700	-2.17999600
C	2.78123800	-0.12043300	-0.67908400
H	4.49716100	1.64755000	-2.85017000
H	1.58858500	1.48240200	0.34363700
C	6.09456600	1.89053500	0.01621100
H	6.94551900	2.19376900	-0.59637800
C	4.57414500	1.83278100	1.68648800
H	4.02994600	2.09107900	2.59549800

C	6.24676100	0.51922400	0.73231600
C	5.34943300	0.49910700	1.72559100
H	5.15696700	-0.31039300	2.41855500
C	2.17223200	-1.32716000	-0.01302100
H	2.16266700	-1.16097600	1.07260900
C	0.69887400	-1.55241700	-0.42433100
C	2.96249600	-2.53649100	-0.26540700
O	0.32299400	-2.61197700	-0.88820200
O	-0.04440500	-0.50965100	-0.19530400
N	3.61474000	-3.46938100	-0.45800900
O	-4.86550000	1.28081600	-1.35651300
Ti	-4.27535500	-0.45686600	-1.65244600
Ti	-1.95815200	-0.17878500	-0.19437500
O	-2.62915900	-1.23568400	1.14318700
O	-2.45994300	-0.58831400	-1.89645500
O	-2.39886300	1.52168500	0.24303500
O	-5.10384800	-1.62904000	-0.47255000
Ti	-4.44799200	-1.17372400	1.14574400
O	-5.14549100	-2.29504300	2.41341300
Ti	-4.22964200	1.75820400	0.25990000
O	-4.67605000	3.49327700	0.63253900
O	-4.86876200	0.59061100	1.49856600
H	-4.78664900	-2.39111800	3.30751500
H	-4.56693600	3.90337700	1.50296000
H	5.44474100	3.77164200	1.01499000
H	1.86012800	1.66646300	-2.31794800
C	5.75545100	2.75339700	1.26350300
C	2.61971700	2.01800700	-1.61439500
H	2.61741800	3.10977200	-1.56203300
H	6.56318000	2.76943300	1.99999900

C	7.17735200	-0.50815800	0.28974900
H	7.90849100	-0.22648800	-0.47864100
N	7.15045300	-1.69620600	0.76466400
N	8.11599400	-2.57335200	0.34565400
H	7.75636800	-3.51972800	0.37864100
H	8.49764900	-2.36524700	-0.57789100

System 5:

C	0.67596400	-0.20848500	1.14932600
C	1.07945900	-0.63488200	2.56028400
C	2.25980000	1.10863700	2.12030800
C	1.40620600	0.86783700	0.88151600
C	2.53220200	-1.14392700	2.46885200
H	2.84700700	-2.17527200	2.56324400
C	3.28279300	-0.06918800	2.18530800
H	0.38902000	-1.21072700	3.17196800
H	2.62723900	2.10598200	2.33977900
C	1.28255600	1.08550900	-0.62991300
H	2.07609800	1.62475700	-1.14260800
C	0.10337300	-0.65034300	-0.20122200
H	-0.18210400	-1.69147800	-0.33619300
C	-0.16781900	1.50784200	-0.90362100
C	-0.91754200	0.43309800	-0.64221100
C	4.68591500	-0.08100300	1.89364300
H	5.21775400	-1.00403300	2.10914600
C	6.87672600	0.67779900	1.07411400
C	5.43750700	0.90309900	1.34617900
O	7.43780300	-0.40736100	1.37423600
O	7.55445700	1.59649900	0.51087300

C	4.90451700	2.16775400	0.94384500
N	4.46229900	3.18413500	0.60865200
H	-0.47957700	2.51007800	-1.16541200
O	1.21294600	-0.29829500	-1.05843700
O	1.33831600	0.66127600	3.13014400
O	10.22378300	-0.76738200	1.44446400
Ti	9.25253000	0.37759400	0.36188100
Ti	11.70671600	1.25830700	-0.68902700
O	11.47116400	0.55412700	-2.38325000
O	10.24160700	1.90530600	0.17838100
O	12.79274500	0.20368900	0.35743200
O	8.91414800	-0.45454900	-1.21506800
Ti	10.47288400	-0.92333500	-2.06343500
O	10.15018600	-1.68645900	-3.69649600
Ti	11.78167400	-1.27214500	0.67181700
O	12.74806700	-2.38347400	1.75904100
O	11.36350300	-2.05647000	-0.93911000
H	10.31770100	-1.29175800	-4.56353600
H	13.68383400	-2.30073800	1.98922700
N	-6.57848800	-0.22680800	-0.71801600
C	-7.16016000	-1.52122800	-0.68791300
C	-6.64723600	-2.54703700	-1.49170100
C	-8.26214600	-1.77314100	0.13956300
C	-7.21020300	-3.81723600	-1.47017600
H	-5.80058900	-2.33995300	-2.13704300
C	-8.83301000	-3.03293500	0.14424600
H	-8.65351200	-0.97593300	0.76385500
C	-8.30925700	-4.05972900	-0.65340000
H	-6.79756100	-4.59964900	-2.10016900
C	-7.42424000	0.91190600	-0.77813700

C	-7.16710200	2.02733700	0.02982300
C	-8.52413100	0.92217500	-1.64489600
C	-7.99828500	3.12984000	-0.05221500
H	-6.31909600	2.01298700	0.70733200
C	-9.36964000	2.02287400	-1.71018600
H	-8.71085400	0.05625900	-2.27053400
C	-9.10242400	3.13247400	-0.91595200
H	-10.21772700	2.01115000	-2.38837300
C	-9.79704300	4.41835900	-0.77975700
C	-10.93413400	4.91180600	-1.41083200
C	-9.11144700	5.19137900	0.16903500
C	-11.38111400	6.18983300	-1.08397900
H	-11.46722800	4.31566800	-2.14577400
C	-9.56083900	6.46221700	0.49127500
C	-10.70118400	6.95963100	-0.14105700
H	-12.26675000	6.59006700	-1.56793900
H	-9.03714200	7.06920200	1.22478000
H	-11.06159100	7.95421700	0.10296900
C	-9.10594000	-5.27272900	-0.43309400
C	-10.11100600	-4.97668300	0.49972000
C	-8.99689400	-6.54714700	-0.97949100
C	-11.01192300	-5.95465100	0.89043800
C	-9.90515500	-7.52622000	-0.58397800
H	-8.21957700	-6.77995300	-1.70143900
C	-10.90485900	-7.23390000	0.34295600
H	-11.79414100	-5.73516900	1.61207500
H	-9.83379400	-8.52602200	-1.00116500
H	-11.60561900	-8.00764000	0.64110100
C	-5.17653500	-0.07031300	-0.70072500
C	-4.56367600	0.96362600	-1.42274600

C	-4.36301800	-0.94218900	0.02976400
C	-3.19020200	1.12281600	-1.39850500
H	-5.17352100	1.63918100	-2.01170900
C	-2.98423700	-0.78764100	0.03106700
H	-4.81448200	-1.74608300	0.60004000
C	-2.36632300	0.25379300	-0.66959500
H	-2.74095200	1.92165900	-1.98026100
H	-2.38387400	-1.47896200	0.61458500
C	-7.90453400	4.44103600	0.71714000
C	-8.02539900	4.21434400	2.23264600
H	-7.18324600	3.62041300	2.60207400
H	-8.02162700	5.17079400	2.76512000
H	-8.95165900	3.68781500	2.48019600
C	-6.59434700	5.18046400	0.40040000
H	-5.73158600	4.59978100	0.74227700
H	-6.48807500	5.35185800	-0.67469400
H	-6.56950300	6.15078600	0.90649300
C	-10.02795700	-3.52422700	0.95075500
C	-11.30405400	-2.74963800	0.58338400
H	-11.20486800	-1.69351900	0.85415200
H	-11.50743900	-2.81090100	-0.48961800
H	-12.16761500	-3.15568000	1.11981300
C	-9.76790300	-3.41985200	2.46211500
H	-9.64771100	-2.37364800	2.76122200
H	-10.60840500	-3.83672900	3.02617000
H	-8.86197900	-3.96360200	2.74510300

System 6:

C	6.22356400	0.76881400	-0.20513800

C	5.88856000	2.18995300	0.25019800
C	4.30789100	0.78986200	1.08754100
C	5.29846500	-0.05035300	0.28769100
C	4.55534300	2.55254200	-0.40861000
H	4.41890000	3.32081300	-1.16154500
C	3.60424400	1.72850000	0.07411000
H	6.66881400	2.94818100	0.19189000
H	3.66232000	0.28244700	1.79966800
C	5.61203200	-1.46237300	-0.21620500
H	4.81517500	-2.20453400	-0.15932500
C	7.17183000	-0.06617600	-1.06815900
H	7.79393900	0.46440400	-1.78724100
C	6.96427300	-1.87916800	0.38520200
C	7.89492600	-1.03780400	-0.10148400
C	2.23665300	1.75363900	-0.36067500
H	1.96231400	2.57553000	-1.01804000
C	-0.13982900	1.18157900	-0.66437900
C	1.21808300	0.90456700	-0.08745500
O	-0.34955900	2.17714700	-1.33853900
O	-1.03303600	0.27386200	-0.36567000
N	9.22178400	-0.88454700	0.26887300
C	9.71070000	-1.85663200	1.22667400
H	9.73510600	-2.87862100	0.81521200
H	10.72188500	-1.58289000	1.53734200
H	9.06507200	-1.86080200	2.10875000
C	10.18944500	-0.54699500	-0.76341700
H	11.12697500	-0.24630300	-0.28834300
H	10.40211200	-1.39118100	-1.43825800
H	9.83558200	0.29171200	-1.36356200
C	1.35541800	-0.26932600	0.71689200

N	1.45775600	-1.22715700	1.36083500
H	7.08323400	-2.63623500	1.14826300
C	6.11875400	-1.06159400	-1.63547600
H	6.56109600	-1.89846500	-2.18488700
H	5.35897500	-0.56505000	-2.24620000
C	5.32198700	1.82747600	1.65856100
H	6.07143900	1.37528500	2.31277300
H	4.83766400	2.67106500	2.16126400
O	-6.11566500	1.44346300	-0.52422600
Ti	-5.31820600	0.25977500	-1.71445800
Ti	-2.93527700	0.01985100	-0.32142300
O	-3.35619500	-1.68428200	0.14021800
O	-3.51307200	0.44387900	-1.99616600
O	-3.58320500	1.08116800	1.01559700
O	-5.87456000	-1.48756400	-1.40579700
Ti	-5.18011600	-1.93153000	0.19571600
O	-5.61204000	-3.66855400	0.58530400
Ti	-5.40689500	0.99889900	1.07396200
O	-6.06992200	2.13367200	2.35067200
O	-5.80082800	-0.75893000	1.44960400
H	-5.37144800	-4.10578800	1.41523100
H	-5.74441700	2.15849000	3.26229300

System 6-D

C	-0.58411800	-0.73679500	-1.20417000
C	-0.88541400	-0.83243900	-2.70203600
C	-2.29062800	0.70664400	-1.79868400
C	-1.40605900	0.16759600	-0.67883100
C	-2.30897800	-1.38007200	-2.82359200

H	-2.57239600	-2.34765800	-3.23594600
C	-3.15710700	-0.48179600	-2.28555000
H	-0.14062600	-1.29620600	-3.34790900
H	-2.81265800	1.64550600	-1.63265300
C	-1.10679200	0.25971800	0.81688400
H	-1.85637500	0.72674600	1.45562100
C	0.29079300	-1.28018500	-0.07604500
H	0.80965600	-2.22440300	-0.24032800
C	0.32623700	0.80152900	0.94783600
C	1.16780400	-0.10236700	0.42685700
C	-4.57162900	-0.69612100	-2.16337600
H	-4.97160900	-1.55410800	-2.69875100
C	-6.92938700	-0.45635800	-1.49627600
C	-5.49839600	-0.00341500	-1.46119700
O	-7.28239500	-1.37190600	-2.22223300
O	-7.71685200	0.21780000	-0.69893500
C	-5.18941400	1.12396000	-0.63790700
N	-4.94572300	2.03354300	0.03724900
H	0.56931300	1.78369700	1.33538200
C	-0.78200000	-1.24682300	1.05428600
H	-0.36884700	-1.44702100	2.04791700
H	-1.63086600	-1.90754600	0.85630000
C	-1.23043800	0.67131300	-2.94130300
H	-0.38237600	1.33504200	-2.75529800
H	-1.65563000	0.86644600	-3.93122600
O	-12.73972500	-1.18412100	-0.42668300
Ti	-11.46283700	-1.65415000	0.83632200
Ti	-9.52351800	0.11372600	-0.04874500
O	-9.73014300	1.11089100	1.45831900
O	-9.71750200	-1.66670500	0.27208200

O	-10.67788600	0.78414200	-1.29379500
O	-11.75254100	-0.80448100	2.46133800
Ti	-11.42934100	0.93916700	2.13936000
O	-11.62195500	1.89230800	3.69143700
Ti	-12.40927000	0.56242500	-0.74150800
O	-13.59270100	1.15632000	-2.00762800
O	-12.55280200	1.51785900	0.82013400
H	-11.50168400	2.85128800	3.75158800
H	-13.57038200	2.04906500	-2.38207500
N	6.83733100	0.20689600	-0.18480300
C	7.47334200	1.47117100	-0.24397800
C	6.88857500	2.52014900	-0.96615200
C	8.69075300	1.68240600	0.41844000
C	7.49420200	3.76902600	-1.02702600
H	5.95147200	2.34730500	-1.48361100
C	9.30049800	2.92116500	0.33954300
H	9.13864300	0.87172500	0.98435400
C	8.70679400	3.96956300	-0.37686100
H	7.02334000	4.56823000	-1.59186400
C	7.60337000	-0.98213800	-0.27269700
C	7.31225500	-2.07018700	0.56161700
C	8.65496200	-1.07917800	-1.19368600
C	8.06400300	-3.22625200	0.45737200
H	6.50180100	-1.99121600	1.27927000
C	9.42032400	-2.23534500	-1.28284200
H	8.86840000	-0.23682200	-1.84241300
C	9.12190800	-3.31437000	-0.45799900
H	10.23156200	-2.28925200	-2.00292100
C	9.73788200	-4.64066000	-0.33177200
C	10.80917400	-5.21729500	-1.00596000

C	9.05119800	-5.35270100	0.66310200
C	11.18872400	-6.51630800	-0.67608700
H	11.34362900	-4.66875600	-1.77616100
C	9.43338200	-6.64448900	0.98823600
C	10.50760900	-7.22526000	0.31253000
H	12.02273700	-6.98063000	-1.19342100
H	8.90800400	-7.20384700	1.75759800
H	10.81516100	-8.23695300	0.55856100
C	9.56945200	5.15232100	-0.27286500
C	10.68450100	4.81737700	0.50987200
C	9.43014700	6.43205300	-0.79968400
C	11.66488400	5.76176000	0.76972300
C	10.41896000	7.37716800	-0.53659000
H	8.56769000	6.69472900	-1.40541500
C	11.52760100	7.04632700	0.24144400
H	12.53176400	5.51259800	1.37577900
H	10.32510600	8.38048800	-0.94072100
H	12.28995800	7.79383900	0.43815500
C	5.43115000	0.12918200	-0.03429900
C	4.68194800	-0.78572100	-0.77645500
C	4.76223400	0.96355900	0.86941800
C	3.30582800	-0.86790300	-0.61358500
H	5.18243400	-1.43598400	-1.48583400
C	3.38832000	0.88496000	1.01515300
H	5.33111000	1.66961600	1.46480800
C	2.62359900	-0.03028700	0.27614100
H	2.75190700	-1.58361900	-1.21206700
H	2.89761500	1.52938600	1.73776900
C	7.92358000	-4.51664000	1.25426200
C	8.13390100	-4.27523000	2.75779600

H	7.35037800	-3.62325600	3.15716600
H	8.09672700	-5.22143400	3.30708600
H	9.10260500	-3.80501300	2.95015700
C	6.55407000	-5.17308300	1.01560900
H	5.74928500	-4.53044500	1.38678300
H	6.38390300	-5.35486900	-0.04951400
H	6.49052500	-6.13103900	1.54152600
C	10.61340400	3.36706200	0.97032800
C	11.79718200	2.54822300	0.43057900
H	11.69743800	1.49516200	0.71290700
H	11.85232600	2.60674700	-0.66032000
H	12.74120000	2.91959900	0.84215400
C	10.56266500	3.26782300	2.50341000
H	10.44977900	2.22554900	2.81857800
H	11.48704300	3.65457000	2.94432400
H	9.72379700	3.84128800	2.90823100
